# Age-appropriate design of smart senior care product APP interface based on deep learning

**DOI:** 10.1016/j.heliyon.2024.e28567

**Published:** 2024-03-25

**Authors:** Si Chen

**Affiliations:** College of fine arts, Sichuan University of Science and Engineering, Zigong, 643000, China

**Keywords:** Deep learning, Smart senior care, Age-appropriate design, APP interface, Deep Q-network algorithm

## Abstract

With the aging of the population, the quality of life and happiness of the elderly are increasingly becoming concerns of society. Smart senior care (SSC) products are an important tool to improve the quality of life of the elderly, and their application has been widely discussed. However, due to differences in cognitive characteristics and habits of the elderly, they may face difficulties when using smart products. Therefore, how to design a suitable SSC product APP interface for the elderly has become an urgent problem to be solved. The rapid development of deep learning (DL) provides a new way to deal with this problem. This paper aims to optimize the APP interface design of SSC products suitable for the elderly by using DL technology. Specifically, this paper designs a model based on a deep-Q-network (DQN) algorithm, which can better adapt to the usage habits and needs of the elderly through training and optimization. At the same time, the proposed model is evaluated comprehensively to verify its performance and superiority. To achieve the above goals, this paper first summarizes the existing SSC technology and the design suitable for the elderly. On this basis, a model based on the DQN algorithm is designed, and four datasets are used for training and testing. In the design of the model, this paper pays special attention to how to make the interface more friendly and easy to operate to meet the specific demands and preferences of the elderly. After a comprehensive evaluation, it is found that the proposed DQN algorithm model has achieved remarkable improvement in performance. This model has an average return of about 8.8 and 3.9 compared to other models, which have an average return of about 0.9 and 0.2, respectively. This shows that the model designed in this paper performs well in terms of accuracy and average return and performs better than other algorithms. The results of this paper reveal that optimizing the age-appropriate design of the SSC product APP interface through DL technology can notably enhance the user experience and satisfaction of the elderly. This not only helps the elderly to make better use of smart products and improve their quality of life but also provides useful inspiration and guidance for research and practice in related fields.

## 1 Introduction

### 1.1 Research background and motivations

With the intensification of the aging trend of the global population, the demand for smart products by the elderly has shown a growing tendency. Smart products are playing an increasingly important role in the daily life of the elderly, from health monitoring and communication to leisure and entertainment, and have a wide range of applications. However, due to the cognitive and physical decline of the elderly, they may face some challenges when using smart products. They may face cognitive difficulties in understanding and mastering complex operations. At the same time, the decline of vision and hearing also increases the difficulty of interacting with smart products. In addition, security risks are also a concern for the elderly when using smart products. These challenges make it difficult for the elderly to enjoy the convenience brought by smart products, and measures need to be taken to optimize and improve. As a vital technology in the field of artificial intelligence (AI), reinforcement learning (RL) has shown broad application prospects in many fields. Deep reinforcement learning (DRL) can solve complex problems by introducing deep neural networks (DNNs) into RL models as a new RL technology. It has been widely used in areas such as image processing, speech recognition, and autonomous driving [1]. Based on this, it is an innovative technology to use DRL technology to improve the difficulties faced by the elderly in using smart products.

As a typical algorithm in DRL, the Deep Q-Network (DQN) algorithm has been extensively applied in many practical problems and has achieved remarkable performance improvements [2]. However, due to the high complexity of the DRL model, the learning process of this algorithm is very time-consuming. Many factors, such as the selection of hyperparameters and the quality of training data also influence the algorithm's performance. As a result, further research is needed to optimize and improve the application of the DQN algorithm in RL problems [3].

The main purpose of this paper is to explore the optimization and improvement methods of the DQN algorithm by comprehensively evaluating and testing its application in RL problems to offer technical support for improving intelligent products for the elderly. The motivation of this paper is to enhance the training efficiency and stability of the DQN algorithm and to provide more reliable tools and methods for solving practical problems in the RL field. By optimizing and improving the DQN algorithm, this paper provides greater help and guidance for the practical application of RL, promotes the development of AI technology in various domains, and affords more effective solutions for solving complex problems. The research in this paper is expected to promote the DQN algorithm's development in the field of DRL, offer a more reliable and efficient algorithm model for solving practical problems, and give reference and enlightenment for the research and application of related fields. This paper aims to optimize and improve the DQN algorithm in the DRL field.

### 1.2 Research objectives

Through experimental evaluation of multiple datasets, the performance of the DQN algorithm with traditional convolutional neural network (CNN) and fully convolutional network (FCN) algorithm models in terms of accuracy and average return. Methods and strategies to improve the performance of the algorithm are also sought. In addition, the coping strategy of the DQN algorithm in a complex environment, the optimization algorithm's training process, and the model's generalization ability are also discussed. Through these studies, it is hoped to improve the application effect of the DQN algorithm in RL problems and promote its application in practical problems.

This paper examines the following aspects. Firstly, experiments are designed to evaluate the performance of DQN algorithms in RL problems and compare their advantages and disadvantages with CNN and FCN algorithms. Secondly, aiming at the problems of the DQN algorithm in complex environments, coping strategies are explored, such as increasing the memory mechanism and introducing experience playback, to improve its performance and stability. Moreover, the optimization training process of the algorithm is studied, and how to select appropriate hyperparameters and training strategies is explored to further enhance the algorithm's performance. Finally, the model's generalization ability is examined, and the adaptability and generalizability of the model in different environments are evaluated.

The innovation and contribution of this paper are mainly reflected in the following aspects. Firstly, in terms of research methods, DL technology is introduced to optimize the APP interface of SSC products. This approach not only improves the personalization and ease of use of the interface but also makes smart products more adaptable to the needs of the elderly. Secondly, the integrity of the experimental design is also a highlight of this paper. Through comprehensive evaluation using four datasets, this paper verifies the performance superiority of the proposed model, which makes the research results more convincing. In addition, the research results of this paper have high practical value. By optimizing the APP interface of SSC products, this paper can improve the user experience and satisfaction of the elderly, and thus improve their quality of life. This is not only a boost to SSC technology but also a positive contribution to the well-being of the elderly. Finally, the research results furnish useful inspiration and guidance for the research and practice in related fields. The application of DL technology in age-appropriate design is not limited to SSC products, but can also be extended to other fields to provide new ideas and methods to solve the problems faced by the elderly. To sum up, the research in this paper has obvious innovation and contribution in methods, experimental design, and practical value.

## 2 Literature review

RL is an important technology in machine learning, which has received widespread attention and application in recent years. As an emerging technology in RL, DRL can solve complex problems by introducing DNNs into RL models and has been widely used in image processing, speech recognition, autonomous driving, and other fields [4].

As a typical algorithm in DRL, the DQN algorithm has received extensive attention and research since it was proposed. The DQN algorithm proposed by Mohammad et al. (2021) achieved good results by introducing CNNs into Q-learning algorithms and applying them to Atari games. Over time, more and more researchers were applying DQN algorithms to problems in different fields, such as robot control and natural language processing [5]. The Prioritized Experience Replay method proposed by Lo et al. (2021) introduced a prioritization mechanism into the DQN algorithm, which could utilize historical experience to improve the learning efficiency of the algorithm [6]. The Dueling Network architecture proposed by Taverniers et al. (2021) further enhanced the learning effect of the DQN algorithm by decomposing the Q-value network into value and dominance functions [7].

In summary, DRL has many kinds of algorithms, each with unique advantages and limitations. Therefore, selecting and adjusting according to the specific problem and application scenario is necessary when selecting an algorithm. Here, the optimization and improvement methods of the DQN algorithm in RL problems are explored. This provides technical support for the optimization of the age-appropriate design of the interface of the Smart Senior Care (SSC) product APP.

## 3 Research methodology

### 3.1 Age-appropriate design elements of the ssc product App interface

**Fig. 1:**
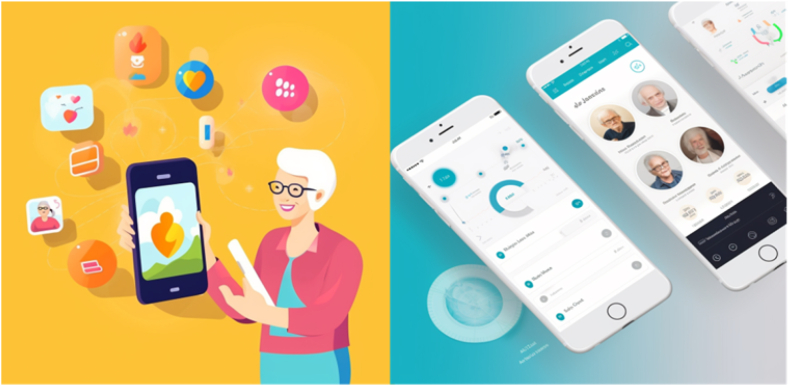
Age-appropriate design principle of the interface of SSC product APP (a is the applicable object, and b is the interface details).

To provide an age-appropriate design of the SSC product APP interface, this paper comprehensively employs a variety of methods, mainly including the following aspects:

First, through user research and feedback mechanisms, the needs and habits of the elderly can be deeply understood, and their direct feedback can be collected to ensure that the design is truly in line with the experience of the elderly [8]. Second, accessibility principles need to be followed. Special attention is paid to vision and hearing problems in the elderly, using design elements such as large fonts, high contrast, and providing audio accessibility features to ensure that the interface is easy to read and understand [9]. Moreover, this paper uses a simple and intuitive interface design to reduce the user's cognitive load and make the main functions and information at a glance. At the same time, DL technology is used for adaptive learning and personalized settings, and the interface and function recommendations are automatically adjusted according to the user's behavior and preferences. This paper also attaches importance to user security and privacy protection, ensuring that APP can comply with relevant regulatory requirements and take appropriate security measures to protect user data [10]. Lastly, through continuous user feedback loop, training and guidance materials, multi-channel interaction design, guided design, and storied interface, as well as A/B testing and iterative optimization, the APP design is continuously optimized and improved to meet the changing needs of users [11]. In summary, through the comprehensive application of these methods, this paper can design an age-appropriate SSC product APP interface to improve the usage experience and satisfaction of the elderly. The age-appropriate design of the SSC product APP interface is displayed in Fig. 1.

In Fig. 1, the SSC product APP is a functional tool and should become an important platform for socialization, entertainment, and leisure for elderly users [12]. Thus, the interface design should reflect certain humanistic care and emotional needs, such as appropriate text, picture, audio, and video elements, friendly social functions, and interactive experiences [13].

In summary, the age-appropriate design elements of the APP interface of SSC products are multifaceted and need to be comprehensively considered from visual, cognitive, and emotional aspects [14]. Only under the premise of fully considering the characteristics and needs of the elderly can the functions and effects of SSC product APP be truly realized so that they can enjoy the convenience of science and technology and improved quality of life [15].

### 3.2 Characteristics and needs of the elderly using the App

In recent years, with the popularity of smartphones and apps, the elderly have gradually begun using apps to facilitate their daily lives. However, the characteristics and needs of the elderly using APP are also different due to some differences in the use of APP by the elderly and young people [16].

First, old people are unfamiliar with the use of technology, which is a major feature of the use of APPs by the elderly. Therefore, it is necessary to provide clear and clear operation guidelines and educational support for the elderly to facilitate their smooth use of the APP. Second, the sensitivity of the elderly to parameters, such as font size and brightness, is another factor that affects the use of APP by the elderly [17]. The eyes of old people may be affected by aging, so they need large font sizes and high screen brightness to use the APP. Hence, the APP should provide options for adjusting font size and brightness to meet the personalized needs of the elderly. Finally, the attention of the elderly to safety is also an essential feature of their use of APP. Old people are sensitive to privacy and security issues, so they need Apps to provide sufficient protection measures. The APP should take various security measures, such as user authentication and data encryption, to ensure the security of old people using the APP [18].

### 3.3 Application of deep learning technology In the age-appropriate design of App interface

With the advent of an aging society, more and more old people are using smartphones and mobile applications [19]. However, many old people may face difficulties because these applications are usually not designed for their specific needs. Therefore, age-appropriate design of app interfaces is becoming increasingly important. Deep Learning (DL) techniques are a powerful tool that can help developers understand the needs of old people and deliver smart, adaptive, and personalized APP experiences [20].

In the digital age, how to let the elderly better integrate and use various applications has become an important issue. Considering the various challenges that the elderly may encounter when using APPs, such as decreased vision, memory, and low acceptance of new technologies, DL technology provides innovative solutions. First, considering that the reading ability of the elderly is often affected by age, adaptive font size and style adjustments have become crucial. By utilizing DL technology, the elderly's reading ability can be evaluated, including but not limited to their visual condition, reading speed, and comprehension ability. According to these evaluation results, the APP can automatically adjust the font size, style, and contrast to ensure that the content is clear and easy to read for the elderly. In this way, they can easily read the content in the APP and avoid eye fatigue caused by long-term reading, thus improving their experience and usage efficiency [21]. Second, considering the inconvenience faced by the elderly in operation, the introduction of gesture recognition technology has become particularly important. Many old people may not be familiar with or forget common gesture operations, but through DL technology, they can recognize and parse their gestures, such as sliding, clicking, etc., and provide corresponding feedback. This means that even if the elderly do not fully remember how to operate the APP, they can easily complete various tasks through gestures, making it more convenient to use the APP [22]. Third, for the elderly who are not good at or accustomed to using touch screens or keyboards, voice assistants become a powerful helper. With DL technology, an intelligent voice assistant system can be built that allows the elderly to control the APP using voice commands. This not only removes them from tedious manual operations but also enables the elderly to complete various tasks without touching the screen, thus greatly improving their experience and efficiency. Fourth, the search function is essential for any APP, but the elderly may encounter situations where the search content does not match the relevant information. This may be because their search methods, vocabulary, or memory are different from those of young people. To this end, DL technology can help more accurately match the search intention and relevant information of the elderly [23]. For example, by analyzing and learning about the search history, interests, hobbies, and other information of the elderly, content that is more in line with their needs can be recommended, thereby promoting their search efficiency and experience [24]. Lastly, for problems that are difficult to describe in words, image recognition technology provides another solution for the elderly. Through DL technology, photos or images provided by the elderly can be matched with relevant information in the APP, providing them with accurate answers. This not only solves the descriptive difficulties that elderly people may face but also enables them to interact with APPs intuitively, improving their convenience and satisfaction in using APPs [25]. To sum up, DL technology plays a crucial role in enhancing the APP usage experience of the elderly. From adaptive font size and style adjustments to the introduction of features such as gesture recognition, voice assistants, intelligent search, and image recognition, these technologies offer a more friendly, convenient, and efficient APP-use experience for the elderly. As technology continues to advance, it is reasonable to believe that the APPs of the future will be more suitable for the elderly to use, enabling them to better integrate into the digital society.

In short, DL technology can play a large role in the age-appropriate design of APP interfaces. It can help developers understand the needs of the elderly and provide an intelligent, adaptive, and personalized APP experience. In the future, with the intensification of aging, APP age-appropriate design will become more important. Also, DL technology will continue to provide opportunities to make breakthroughs in this field.

### 3.4 Dl App interface age-appropriate design model

**Fig. 2:**
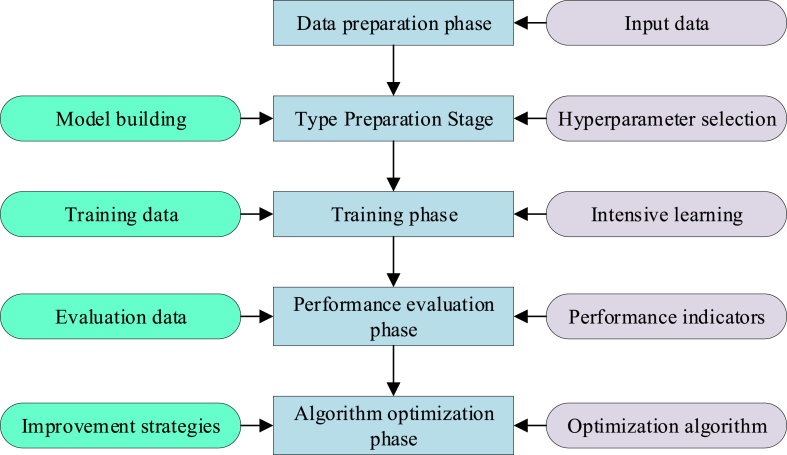
Computational code of DL APP interface age-appropriate design model.

In terms of the origin and development of research methods, DL and RL are among the most active and important branches in the field of AI in recent years. DL has its roots in the study of artificial neural networks, while RL can be traced back to its inspiration in behavioral psychology. With the improvement of computing power and the emergence of big data, DL and RL have made significant breakthroughs and applications in many fields [26]. In the field of APP interface design, the application of DL and RL is also getting more and more attention. These methods can be used to understand and predict user behavior and make adaptive adjustments based on user feedback to optimize interface design and user experience. By training the model to extract useful features from large amounts of data, this paper can better understand user needs and preferences and design a more suitable interface. In previous studies, some researchers have explored how to use user behavior data to train predictive models to predict user preferences for different interfaces. These studies prove the validity of data-driven methods in interface design and provide a basis and reference for further research. These experiences not only help this paper to better understand the needs and behaviors of users but also offer useful guidance and inspiration. Specifically, the goal of this paper is to combine DL and RL technology to design an APP interface suitable for the elderly. The behavior and preferences of the elderly can be understood and predicted through DL. Through RL, this paper can make adaptive adjustments according to the feedback and behavioral data of the elderly and optimize the interface design. This research method can improve the convenience and satisfaction of the elderly and provide useful guidance and enlightenment for research and practice in related domains. In general, DL and RL, as two important branches in AI, have broad application prospects in the field of APP interface design. By combining these technologies, the needs and preferences of the elderly can be better understood and adjusted based on their feedback, further improving their satisfaction and usage experience. This research method is helpful in improving the quality of life of the elderly and can give useful guidance and enlightenment for research and practice in relevant fields. DRL is a method that combines DL and RL to enable autonomous learning and decision-making in complex environments. DRL algorithms can be used when designing DL APP interface age-appropriate design models [27]. Fig. 2 suggests the computational code for implementing DQN using TensorFlow in this paper.

Fig. 2 demonstrates that the design of the age-appropriate model of the APP interface using DL technology is realized [28]. The calculation flow of DQN is as follows [29].

First, the objective Q-value function is defined.

(1) $$Q_{target}\left(s,a\right)=r+\gamma \max_{a^{\prime }}Q_{target}\left(s^{\prime },a^{\prime }\right)$$

In Eq. (1), s represents the current state; a refers to the current action; r means the reward obtained after executing the action $a^{\prime }$; γ stands for the discount factor, and $s^{\prime }$ indicates the new state [30]. Then, the actual Q-value function is defined.

(2) $$Q_{actual}\left(s,a\right)=r+\gamma \max_{a^{\prime }}Q_{actual}\left(s^{\prime },a^{\prime }\right)$$

In Eq. (2), $Q_{actual}$ is the current Q-value function [31]. Next, the Mean-Squared Error is used as a loss function.

(3) $$Loss=\frac{1}{2}\sum_{i}{\left(Q_{target}\left(s_{i},a_{i}\right)-Q_{actual}\left(s_{i},a_{i}\right)\right)}^{2}$$

Finally, gradient descent is used to update the Q-value function.

(4) $$Q_{actual}\left(s,a\right)=Q_{actual}\left(s,a\right)-\alpha \nabla_{Q_{actual}\left(s,a\right)}Loss$$

In Eq. (4), α refers to the learning rate, and ∇ represents the gradient [32].

DQN is an RL algorithm based on DL. It mainly consists of a DNN and an experience playback mechanism. DQN aims to select the optimal strategy by learning the value function. DNN is the core part of the DQN algorithm, which uses a set of parameters to approximate the Q-Value function. The input to the DNN is a characteristic representation of the current state, and the output is the state-action value for each action. Typically, the structure of a DNN consists of multiple hidden layers, each made up of many neurons that map inputs into a non-linear space via activation functions.

The experience playback mechanism is another important part of the DQN algorithm. It builds an experience playback buffer by storing the experiences the agent has observed in past interactions, including states, actions, rewards, and the next state. In the training process, DQN randomly samples a batch of experiences from the experience playback buffer to reduce the correlation between samples and improve the efficiency and stability of training.

FCN is a commonly used neural network structure called Multi-Layer Perceptron (MLP). In FCN, each neuron has a connection with each neuron in the previous layer, that is, a full connection. FCN implements the mapping relationship between input and output by stacking multiple fully connected layers (FCLs). However, the fully connected structure of FCN may lead to too many parameters and cannot effectively process high-dimensional data such as images, so in image processing, CNNs are often combined to process.

CNN is a special neural network structure, which is widely used in the field of image processing. The main idea is feature extraction and dimensionality reduction through the convolutional and pooling layers to capture features of different levels in the image. In CNN, the convolutional layer uses a set of learnable filters (convolution kernels or feature detectors) to perform convolution operations on the input image, thus extracting different features. The pooling layer is employed to reduce the size of the feature map and preserve the most prominent features. By stacking multiple convolutional and pooling layers, CNN can extract and learn local and global features of images layer by layer.

In summary, DQN is a DL-based RL algorithm, with DNN and experience replay mechanism as key components. FCN is a commonly used fully connected neural network structure, while CNN is a special neural network structure suitable for image processing. They play important roles in various fields and tasks and have different mathematical expressions and calculation methods.

## 4 Experimental design and performance evaluation

### 4.1 Datasets collection

Based on the above design, the dataset is used to comprehensively train, test, and evaluate the designed model, which supports improving the model's performance [33]. The datasets used are as follows.

1) Arcade Learning Environment: an Atari game dataset. It contains multiple game images and action sequences [34].
2) OpenAI Gym: an RL test environment. It provides datasets for several game and problem scenarios, including classical controls, Atari games, and Robotics [35].
3) DeepMind Control Suite: an RL test environment. It is used to test and evaluate the performance of robot control algorithms, which include dozens of tasks using a continuous action space [36].
4) Mujoco environment: a physical simulation environment. It can train and test RL-based control algorithms [37].

### 4.2 Experimental Environment

1) Hardware devices. This experiment uses the NVIDIA Tesla V100 Graphic Processing Unit (GPU) card, which is a high-performance computing accelerator suitable for DL.
2) Operating system and software. A suitable operating system and software environment can support the installation and operation of DL frameworks. Thus, the Ubuntu operating system and Compute Unified Device Architecture DL acceleration library are selected here. In addition, the DL framework TensorFlow or PyTorch is installed [38].
3) Environmental simulator. Here, the OpenAI Gym emulator (version 0.20.6) is selected to simulate the RL environment effectively. OpenAI Gym is a popular RL environment simulator that provides a standardized interface for researchers to develop and compare RL algorithms. With OpenAI Gym, this paper can create and reproduce various RL tasks to evaluate and improve the algorithm. The simulator supports a variety of environments, encompassing continuous and discrete action spaces, which makes it an ideal tool for studying various RL problems. In version 0.20.6 of OpenAI Gym, the emulator has received several improvements and enhancements, including better documentation and examples, performance improvements, and support for new environments. In addition, this release also fixes some known issues and enhances the stability of the emulator. By using the OpenAI Gym simulator, the RL environment can be effectively simulated, providing a consistent and reliable platform for the APP interface design problem studied in this paper. This facilitates developing, testing, evaluating, and optimizing APP interface design algorithms suitable for the elderly [39].
4) DQN algorithm implementation. According to the selected environment simulator and DL framework, the DQN algorithm is implemented, encompassing the DNN structure and experience playback mechanism [40].
5) Training and testing. The model is trained and tested using the selected environment simulator and DQN algorithm, and the performance indicators of the model are recorded.
6) Performance optimization. Different DQN algorithm optimization strategies are tried, such as tuning hyperparameters and using other algorithms, such as Double DQN, to improve the model's performance.
7) Hardware optimization. Various hardware optimization strategies are tried, such as using multi-GPU cards for parallel computing and FP16 precision for calculation, to improve the speed and efficiency of model training and testing.

### 4.3 Parameters setting

This paper mainly introduces how to use DL technology to optimize the APP interface design of SSC products suitable for age. A model based on the DQN algorithm is designed to achieve this goal. The main idea of the model is to understand the usage habits and needs of the elderly through training and learning, thus providing them with a more personalized and friendly interface. This paper uses four datasets for a comprehensive evaluation to verify the performance superiority of the proposed model. In the process of implementing the model, this paper first analyzes user behavior to understand the usage habits and preferences of the elderly. Then, based on these data, this paper constructs a DQN model for learning the interaction patterns and decision-making processes of the elderly. In the model's training process, this paper adopts the experience playback technology to train the model to learn by constantly collecting the interactive data of the elderly when they use the SSC product APP. At the same time, this paper also uses multi-thread processing technology to improve the efficiency and stability of training. After training and optimization, the model can adapt to the habits and needs of the elderly and provide more intelligent services. Furthermore, the model can also be continuously optimized and enhanced according to the feedback and behavior of the elderly, and constantly improve the user experience and satisfaction of the elderly. In summary, the DQN-based model proposed here effectively optimizes the APP interface design of age-appropriate SSC products. Through training and learning, the model is able to understand the usage habits and demands of the elderly and provide them with a more personalized and friendly interface. This is conducive to improving the quality of life of the elderly and provides useful inspiration and guidance for research and practice in relevant fields. The DNN structure and number of parameters of the DQN algorithm depend on the problem's complexity and the training data's size. Consequently, the specific parameters must be determined through continuous training. The following are the initial parameters of the design in this paper.

The first is the input layer. The number of neurons in the input layer usually equals the state space dimension. A value of 210 × 160 pixels can represent the state, so the number of input layer neurons is 210 × 160 = 33,600.

The second is the convolutional layer. The convolutional layer's parameters include the convolution kernel's number, size, and step size. The convolution kernel size is 3 × 3 or 5 × 5, and the number of convolution kernels is usually 32 or 64.

The third is the pooling layer. The parameters of the pooling layer include the size and step size of the pooling kernel. The pool kernel size is 2 × 2, and the step size is 2.

The fourth is the FCL. The number of neurons in an FCL is usually determined based on the problem's complexity and the training data's size. The number of FCL neurons is 512.

The fifth is the output layer. The number of neurons in the output layer is usually equal to the dimension of the action space. The dimension of the action space is the number of actions to choose from. The experiment involves only two actions (up or down), so the number of neurons in the output layer is two.

In addition to the above basic parameters, the DQN algorithm also involves some hyperparameters, such as learning rate, discount factor, and experience playback cache size. To achieve optimal performance, these parameters must be optimized through cross-validation and other methods.

1) Learning rate. The value in the example is 0.00025.
2) Discount factor. The value in the example is 0.99.
3) Exploration rate. One is set at the start of training. Then, it gradually decreases according to the exponential decay function until the minimum value is 0.1.
4) Experience playback cache size. The value is between 10,000 and 1 million, depending on the training data's size and the problem's complexity.
5) Batch size. The value in the example is 32 or 64.
6) Target network update frequency. The target network is typically updated every 1000 iterations.

### 4.4 Performance evaluation

**Fig. 3:**
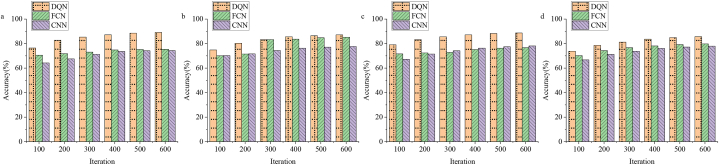
Model accuracy evaluation results (a: Arcade Learning Environment dataset, b: OpenAI Gym dataset, c: DeepMind Control Suite dataset, and d: Mujoco environment dataset).

A dataset is applied to train and test the model to evaluate the overall performance of the proposed model. Furthermore, the model designed here is compared with the FCN and CNN models to explore the performance level of the model. Fig. 3 plots the results of evaluation of the model.

Fig. 3a, b, 3c and 3d denote that the designed model is comprehensively evaluated through four datasets. It is found that the accuracy of the designed DQN algorithm model is the highest about 87%, and the lowest about 75%, which achieves a tremendous basic breakthrough compared with the CNN and FCN algorithm models. Moreover, the average return of the model is tested and evaluated.

**Fig. 4:**
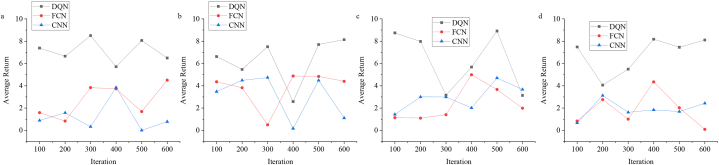
Model average return test results (a: the Arcade Learning Environment dataset, b: the OpenAI Gym dataset, c: the DeepMind Control Suite dataset, and d: the Mujoco environment dataset).

Fig. 4 reveals the results of the average return of the model.

Fig. 4a, b, 4c and 4d present that the average return of the model is tested and evaluated. Through testing, it is observed that the designed model achieves a significant performance improvement compared to other models. The average return of the designed DQN algorithm model is the highest, around 8.8, and the lowest is approximately 3.9, while the average return of other models is the highest, about 0.9, and the lowest is approximately 0.2. It can be found that the DQN-based model is proposed, which effectively optimizes the SSC product APP interface design and improves the user experience of the elderly. Through DL, the model understands the usage habits and preferences of the elderly and intelligently adjusts the interface elements. In addition, the model has the ability to adapt and continuously optimize the interface design to meet the changing needs of users. At the same time, it supports multi-modal interaction to adapt to the requirements of different elderly. In a word, the model can improve the quality of life of the elderly, provide a more friendly interface, and afford useful guidance for research in related fields.

### 4.5 Discussion

In today's society, with the continuous progress of science and technology, intelligent products have become a significant component of the life of the elderly. However, due to the gradual physical and cognitive decline of the elderly, they may encounter many difficulties when using smart products. Therefore, this paper proposes a DQN-based algorithm model to optimize the elderly's experience of using smart products.

Initially, the challenges faced by the elderly when using smart products are analyzed in depth. Due to deterioration in vision, hearing, memory, etc., the elderly may have difficulty understanding and operating smart products. At the same time, they may lack the experience and skills to use smart products, resulting in the inability to make full use of the product's functions. Therefore, from the perspective of user experience, smart products that are more user-friendly and easier to operate should be designed. Subsequently, an algorithm model based on DQN is proposed to optimize the experience of smart products for the elderly. Through DL and RL technology, the algorithm model can automatically learn and optimize the product's operation process to adapt to the habits and needs of the elderly. Moreover, the algorithm model can also adapt to the feedback and behavioral data of the elderly, and constantly optimize the performance and user experience of the product. The experimental section employs four different datasets to test the algorithm model's performance.

The experimental results reveal that the algorithm model has achieved remarkable accuracy and average return outcomes. The highest and lowest accuracies are about 87% and 75%, respectively, indicating that the algorithm model can more accurately predict and classify behavioral data of the elderly. The highest and lowest average returns are around 8.8 and 3.9, which are significantly better than other models, illustrating that the algorithm model has good application prospects in improving the user experience of smart products for the elderly. Finally, a thorough analysis and discussion of the experimental results are conducted. The findings denote that the algorithm model performs well in handling complex problems and massive data. Meanwhile, based on the experimental result analysis and discussion, the following improvement suggestions are proposed to enhance the algorithm model's performance and user experience.

(1) Multidimensional data collection: When studying the APP use behavior of the elderly, data can be collected from multiple dimensions, such as physiological data (such as heart rate, blood pressure, etc.), psychological data (like emotional state, cognitive ability, etc.) and behavioral data (such as frequency and duration of use, etc.). This allows for a more comprehensive understanding of the elderly's needs and preferences.
(2) RL strategy optimization: When applying the RL algorithm, different strategies and methods can be tried, such as exploration-utilization strategy, Q-learning, SARSA, etc., to find the most suitable strategy for APP interface design for the elderly. In addition, it is necessary to consider incorporating other machine learning algorithms, such as ensemble learning or transfer learning, to improve the model's performance.
(3) Improvement of user feedback mechanism: To better adjust the interface design according to the feedback of the elderly, the user feedback mechanism can be further improved, such as setting more intuitive evaluation indicators and providing more convenient feedback channels. This helps increase the elderly's engagement and get more accurate data.

To sum up, the research findings can verify the DQN algorithm's effectiveness in optimizing the use experience of smart products for the elderly and provide useful guidance and inspiration for the research and practice in related fields. In the future, how to apply the algorithm model to more smart products can be further explored to improve the elderly's quality of life and user experience. In addition, it can be further studied how to combine the specific needs and characteristics of the elderly to design a more user-friendly and easy-to-operate intelligent product interface and functions. Compared to Ren et al. (2022) [41] and Wang et al. (2022) [42], the DQN algorithm model designed in this paper exhibits significant advantages in accuracy and average return, and has made fundamental breakthroughs in comparison with CNN and FCN algorithm models. Compared to other studies, the designed model can more accurately predict and evaluate data, and achieve higher average returns.

## 5 Conclusion

### 5.1 Research contribution

By incorporating DL and RL technology, this paper proposes an APP interface design model suitable for the elderly. The model can understand and predict the behaviors and preferences of the elderly from massive historical data, and adaptively adjust based on their feedback and behavioral data to optimize interface design and user experience. The results reveal that the model performs well in the simulated environment and can improve the convenience and satisfaction of the elderly. In this paper, DQN in RL is deeply investigated. The experimental evaluation of four datasets demonstrates that the algorithm has achieved excellent performance regarding accuracy and average return. It indicates that the APP interface design model based on DL and RL provides an effective solution to solve the difficulties faced by the elderly when using APPs. By integrating DL and RL technologies, the model is able to better understand and forecast the elderly's preferences and behaviors and make adaptive adjustments according to their feedback and behavioral data to optimize user experience and interface design. This paper contributes to improving the quality of life of the elderly and making it more convenient for them to use APPs to obtain information, socialize, or enjoy other digital services. Furthermore, it also provides useful guidance and enlightenment for research and practice in related domains. Research contributions are mainly reflected in the following aspects.

1) The DQN algorithm is studied in depth. The DQN algorithm is deeply explored in this paper, and its principle, optimization method and similarities and differences with other DL algorithms are analyzed. The DQN algorithm's theoretical analysis and experimental research provide important reference and guidance for the further research and application of this algorithm.
2) Experimental evaluation of the DQN algorithm performance is carried out. The experimental evaluation of the DQN algorithm on four different datasets shows that the algorithm has achieved excellent performance in accuracy and average return. This gives experimental data support for further research and application of this algorithm and provides a reference for other algorithms in RL.
3) The application value of the DQN algorithm in RL is discussed. The DQN algorithm is a vital method in RL. Experimental evaluation denotes that this algorithm can deal with complex problems and high dimensional data and can be trained and optimized effectively by the DL method. This offers a new idea and direction for the DQN algorithm's application in RL. Moreover, it also provides an important reference for further extension of the RL algorithm in practical applications.

## Future works and research limitations

### Future research directions

Although this paper has achieved promising results, there are still several areas for future research to improve the DQN algorithm. Therefore, future research can evaluate algorithms at different levels of complexity by testing them in a broader dataset. This will help people understand the universality of algorithms and their ability to handle complex problems.

Another area for future research is to explore the impact of different factors on the performance of the DQN algorithm, such as the selection of neural network structure and the adjustment of hyperparameters. This will help improve the robustness and generalization ability of the algorithm.

### Research limitations

One limitation of the research is that the algorithm's performance is only evaluated through experiments without delving into its internal working principles. Consequently, future research can explain and understand the behavior and performance of algorithms by further exploring the theory of algorithms.

Another limitation is that this paper only uses one DL algorithm, the DQN algorithm, to handle RL problems. Thus, future research can explore the application of other DL algorithms in RL and compare and analyze them to understand and improve the performance of RL.

## CRediT authorship contribution statement

**Si Chen:** Writing – original draft, Validation, Software, Resources, Formal analysis, Data curation, Conceptualization.

## Declaration of competing interest

The authors declare that they have no known competing financial interests or personal relationships that could have appeared to influence the work reported in this paper.
